# Resistive Heater Element Based on a Conductive Line in AlN Ceramic Fabricated by Laser Processing

**DOI:** 10.3390/ma18122861

**Published:** 2025-06-17

**Authors:** Nikolay Nedyalkov, Nadya Stankova, Fatme Padikova, Stefan Valkov, Genoveva Atanasova, Tina Dilova, Lyubomir Aleksandrov

**Affiliations:** 1 Institute of Electronics, Bulgarian Academy of Sciences, 72 Tsarigradsko Chaussee, 1784 Sofia, Bulgaria; nestankova@yahoo.com (N.S.); f.padikova@yahoo.com (F.P.); stsvalkov@gmail.com (S.V.); 2 Center of Competence “Smart Mechatronic, Eco-and Energy-Saving Systems and Technologies”, Technical University of Gabrovo, 5300 Gabrovo, Bulgaria; 3 Department of Mathematics, Informatics and Natural Sciences, Technical University of Gabrovo, 4 H. Dimitar Str., 5300 Gabrovo, Bulgaria; 4 Institute of General and Inorganic Chemistry, Bulgarian Academy of Sciences, Acad. G. Bonchev Str., bl. 11, 1113 Sofia, Bulgaria; genoveva@svr.igic.bas.bg (G.A.); tina@svr.igic.bas.bg (T.D.); lubmir@svr.igic.bas.bg (L.A.)

**Keywords:** laser structuring, ceramic processing, integrated resistive heater

## Abstract

The purpose of this work is to demonstrate that laser-induced conductive tracts in AlN ceramic can be applied for fabrication of an integrated resistive heating element. Nanosecond laser processing at a wavelength of 1064 nm of ceramic in vacuum is used for a formation of conductive areas. It is demonstrated that the applied laser fluence and the number of pulses influence strongly the electrical properties of the material in the irradiated zone. The resistance value of the produced tracks with a length of about 4 mm and width of about 1 mm may vary from 17 to about 2000 Ohms, depending on the processing conditions. The material in the processed zone is characterized by means of surface composition, morphology, and electric properties. It is found that the electrical conductivity of the formed structure is based on the ceramic decomposition and formation of aluminum layer. The analysis of the influence of the temperature on the electrical resistance value shows that the material’s conductivity could be preserved after annealing, as in the present study it is confirmed up to 300 °C. The ability of the formed tracks to serve as a basis element of ceramic integrated resistive heater is studied by applying DC voltage. It is found that the fabricated element can be used with a high reliability to about 90 °C without special requirements for contact design and encapsulation. Operation at higher temperatures is also demonstrated as the maximal one achieved is about 150 °C at 10V. The performance of the heater is investigated and discussed as the operation range is defined. The proposed element can be a basis for a design of an integrated heater in ceramic with high stability and applications in everyday life and research.

## 1. Introduction

AlN ceramic is a type of synthesized material with a large number of properties useful for industrial applications [1,2,3]. It expresses high thermal conductivity, comparable to that of metals, high temperature, and chemical stability [4,5,6]. On this basis it is widely used in fabrication of machine parts that face high temperature and temperature gradients [7], and even in aerospace engineering [8]. Furthermore, in combination with its high electrical insulator properties, AlN ceramic is applied as a heat conductor that facilitates cooling in high-power electronics components [9,10]. The material also expresses a high hardness and is applied in cutting tools and bearings. It is also found that transparent AlN ceramics can be fabricated, aiming at optical applications [11]. A specific property of AlN is that it undergoes decomposition under high temperature [12,13]. Even different decomposition reactions can be realized depending on the heating scenario, the result of these reactions easily can lead to formation of an aluminum layer. Thus, if this process can be controlled efficiently, on the surface of the ceramic, which is an excellent insulator, conductive areas can be formed. This structure would have potential in microelectronics where the fabrication of conductive tracks requires multistep processing and use of hazardous chemicals and potential environment pollutants. Due to their ability to concentrate high energy, which practically can modify any material and easily achieve the high spatial resolution of processing, the application of lasers is an efficient way to induce spatially controlled processing of hard and brittle materials [12,13,14]. It is demonstrated that laser processing can induce spatially controlled decomposition in AlN [15,16,17,18,19,20] and formation of conductive areas. The processing conditions strongly affect the process as the resistance of the fabricated tracks can vary by orders of magnitude [20]. The key parameters are found to be the laser fluence, pulse overlapping (in scanning conditions), pulse duration, and air pressure [21,22,23]. For each of these parameters there exists a processing window where a conductive material, with resistance in the range of tens of Ohms for tracks with a length of a few mm, can be formed. The performed works indicate that laser processing in vacuum leads to a lower resistance of the formed areas at fixed other conditions, as also conductive structures with more complex geometries can be fabricated [21,23]. It is also demonstrated that with the decrease in the laser pulse duration, the resistance of the irradiated areas increases, as the use of ultrashort laser pulses results in processing without the formation of a conductive material [14,24]. The mechanisms that define the resistance value of the fabricated material are a complex interplay between different chemical reactions that include AlN decomposition, oxidation, nitration, and morphology changes that influence the conductive layer spatial characteristics [19,21,25].

Although the process of laser-induced formation of conductive areas has been studied by different groups under different processing conditions, the presented works so far do not show the application of the structures. Such a direction, however, is important since AlN ceramic has found use in different areas, and the formation of conductive lines could additionally expand the potential application and could reduce production costs when, for example, a complex electrical circuit on AlN ceramic is designed. Furthermore, information about the thermal stability of the conductive tracks is also not presented so far, which is, however, crucial, since the material is often used in elevated-temperature conditions.

In this work we present an idea of a new application of AlN in the field of resistive heating element development. The purpose of this research is to demonstrate that by simple laser processing, an integrated heating element with a potential broad range of applications can be fabricated. The efficiency related to processing time and reproducibility defined by the laser method, and the specifics of the used material as one with a high thermal conductivity, determine an attractive potential application of such an element in annealing, heating, and ensuring optimal working conditions for gas sensors and optical elements where the operational temperature is above room temperature. Presenting the electrical properties in terms of resistance, in passive mode where a conductive track is externally heated, and in active mode, where it is used as a resistive heater, the applicability and the operational range for such an application are defined. Furthermore, this work gives new data about the influence of the temperature on the electrical properties of the studied structures, which is an important characteristic of the formed material not only for heater application.

## 2. Materials and Methods

**Figure 1:**
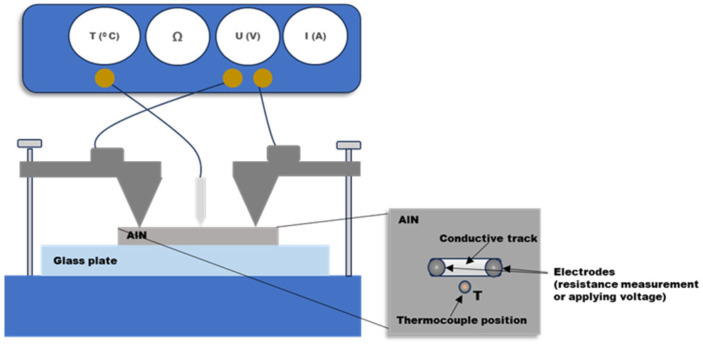
Scheme of the experimental setup for measurement of the fabricated track resistance and temperature. When heater properties are studied, DC voltage is applied on the electrodes and the temperature is measured by thermocouple.

AlN ceramic slabs (Ceram Tec, Plochingen, Germany) with a thickness of 0.6 mm were used in the experiments. The samples were placed on an X-Y computer-controlled stage which allowed fabrication of structures with different geometries and scanning conditions. A Nd:YAG laser system (LotisTii LS-2147, Minsk, Belarus) operating at a wavelength of 1064 nm and a pulse duration of 15 ns is used for processing. The pulse repetition rate is 10 Hz. The laser radiation is focused by a quartz lens with a focal distance of 20 cm in a spot with diameter of about 1 mm, i.e., the sample is before the lens focal point. The spot size is estimated by measuring the modified area on thermosensitive paper. The fabricated tracks have a length of 4 mm. Experiments with different velocities of the translation stage were performed in order to produce tracks with different values of pulse overlapping, *p* = *d.f*/*v*, where *d*, *f*, and *v* are the laser spot diameter, pulse repetition rate, and stage velocity, respectively. Experiments at different air pressures were also performed, and in these cases a mobile vacuum chamber was used. During the processing it is fixed on the translation stage. The morphology and composition of the modified areas were analyzed by a Scanning Electron Microscope (SEM) (Inspect F50, FEI, Hillsbro, MD, USA), equipped with Energy Dispersive X-ray (EDX) (Quantax 200 Bruker, Billerica, MA, USA) spectroscopy. X-ray Photoelectron Spectroscopy (XPS), (AXIS Supra, Manchester, UK) with standard convolution software (ESCApeTM of Kratos Analytical Ltd., Manchester, New Hampshire, UK)) was also performed in order to estimate the chemical state of the modified surface. The phase composition of the samples was determined using X-ray diffraction (XRD) analysis (Bruker D8 Advance diffractometer, CuKα radiation, Billerica, MA, USA) using the Match! v.3.16 program and the ICDD PDF-2 database (2021) as referent files. The resistance of the formed tracks was measured by the two-probe method using a Shneider IMT 23202 multimeter system (France). Two steel electrodes were attached to the ends of the fabricated track and good contact was ensured by screws. The same system was used for measurement of the temperature of the sample when its resistive heater properties were analyzed. In these experiments, DC voltage was applied between the electrodes and the temperature was measured. The system under consideration here was a ceramic sample with an area of 1 × 1 cm^2^. The thermocouple used was placed about 1 mm from the laser-fabricated track. The ceramic slab was placed on glass substrate with a thickness of 3 mm, which ensures a low dispersion of the heat. A scheme of the experimental configuration is presented in Figure 1.

The thermal stability of the fabricated tracks was analyzed with the ceramic sample placed on a heater and the resistance value at different temperatures measured in air. This was conducted using a homemade heater with a controller where the heating/cooling rate can be tuned. The experiments presented here were conducted at a heating/cooling range of 300 C/min. The presented data were obtained on the basis of three measurements and the standard deviation is presented in the dependences below.

## 3. Results and Discussion

**Figure 2:**
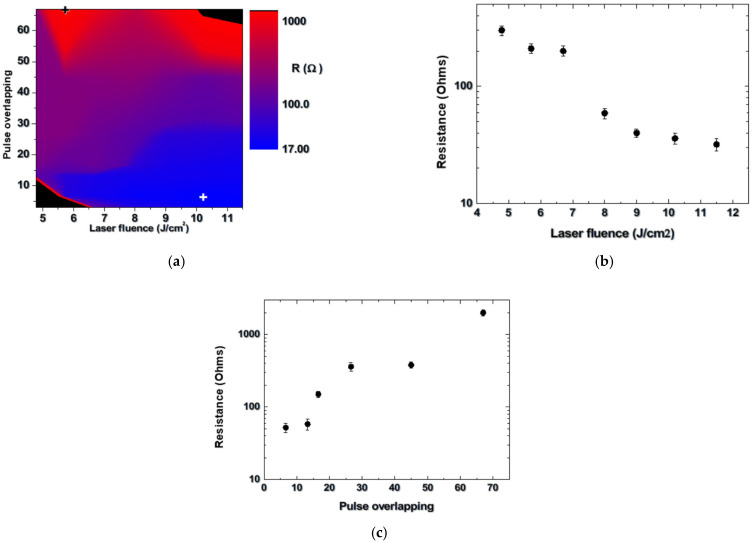
(**a**) Dependence of the resistance of the fabricated structures as a function of the laser fluence and pulse overlapping for laser processing of AlN in vacuum. The black areas represent conditions where the resistance value exceeds the maximum that can be measured with the available system. The white and black crosses indicate the conditions where the minimal and the maximal resistance values are obtained. (**b**) Dependence of the resistance on the laser fluence at a fixed overlapping of 6. (**c**) Resistance dependence on the pulse overlapping at a fixed fluence of 5.7 J/cm^2^.

Our previous experiments [14] show that there is a processing window related to the applied laser fluence and the pulse overlapping where the laser-formed areas are conductive. Furthermore, it is demonstrated that ablation in vacuum results in a reduction in the resistance under fixed other experimental conditions. Considering a heating element, the lower resistance (R) would define an ability of conducting a higher current (I), which leads to a higher heat energy (P) dissipated according to the relation P = I^2^R. On this basis, the study presented here is focused on structures obtained in vacuum (3 × 10^−4^ Torr). Figure 2a shows a dependence of the resistance value of formed tracks on the surface of AlN ceramic on the applied laser fluence and pulse overlapping.

For the presented conditions, a variation of R from 17 to about 2000 Ohms is observed. The areas in black show conditions where the resistance value is higher than the maximal one that can be measured with the available measuring system, which is 2 GOhms. The white and black crosses mark the conditions where the minimal and the maximal values of the resistance are measured, i.e., 17 Ohms and 2000 Ohms, respectively. The former is obtained at a fluence of 10.2 J/cm^2^ and pulse overlapping of 6. The highest value is obtained at a fluence of 5.7 J/cm^2^ and pulse overlapping of 67. For a better quantitative presentation, two examples of the dependences of the resistance on the laser fluence at a fixed overlapping of 6 in Figure 2b, and on the pulse overlapping at a fixed fluence of 5.7 J/cm^2^ in Figure 2c, are also shown.

The presented data indicate that the pulse overlapping of only a few pulses is sufficient for fabrication of a track with resistance in the range of tens of Ohms. This is important since it expresses the ability of fast-track fabrication, i.e., the use of a high translation speed. At low pulse overlapping and low fluence, the decomposition of the nitride ceramic is not efficient and the formed structures are not conductive. Such structures are also produced when the pulse overlapping is in the range of a few tens, especially at the highest fluences used. Note that an experiment conducted in air indicates that at a fluence of 10.2 J/cm^2^ and pulse overlapping of 6, the track resistance is 130 Ohms, about 8 times higher.

**Figure 3:**
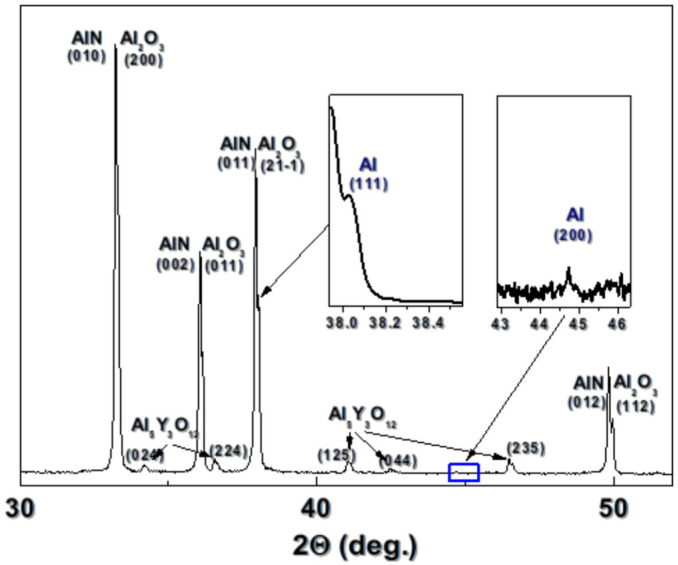
XRD spectrum of laser-fabricated structure on the surface of AlN ceramic after ablation at a fluence of 10.2 J/cm^2^ and pulse overlapping of 6, in vacuum.

The conductivity of the fabricated structures is related to the formation of aluminum on the surface of the ceramic. The decomposition of the AlN is thermally induced as the low-required energy reaction leads to the ejection of nitrogen and remaining aluminum, usually in liquid phase [15,19]. The performed XRD analysis confirms the formation of Al. Figure 3 shows the XDR spectrum of a structure fabricated at a laser fluence of 10.2 J/cm^2^ and pulse overlapping of 6, in vacuum. The analyzed area consists of AlN (98-060-8626), Al_2_O_3_ (98-016-9722), Al (98-0169722), and Al_5_Y_3_O_12_ (98-077-4607).

**Figure 4:**
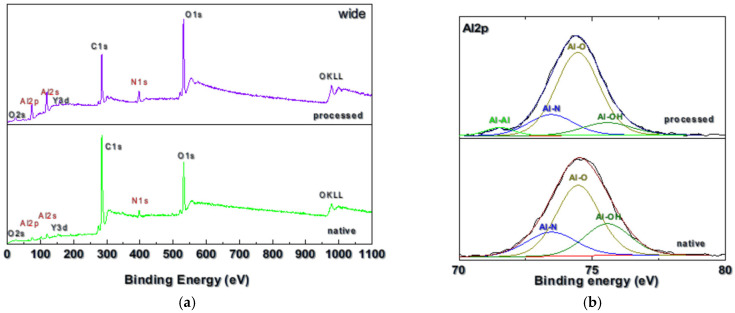
XPS spectra of the native AlN ceramic and after laser processing at a fluence of 10.2 J/cm^2^ and pulse overlapping of 6, in vacuum: (**a**) wide spectrum and (**b**) Al2p.

The presence of the yttrium-composed phase is related to the inclusion of Y_2_O_3_ in the preparation stage of the ceramic, which improves the material stability and formation component. Further clarification of the material composition in the processed area is given by XPS analysis. In Figure 4, wide spectra and Al2p spectra, and their corresponding convolution components for native and laser processed surfaces, are shown. The processing is performed under the same conditions as in Figure 3. The spectra indicate that the surface of the material before and after irradiation is dominated by an oxide phase, and after laser irradiation, metallic aluminum is formed. The surfaces also contain -OH compounds. The wide spectra also show a decrease in the presence of nitrogen (with respect to Al peak intensity), which confirms the realization of the decomposition reaction.

**Figure 5:**
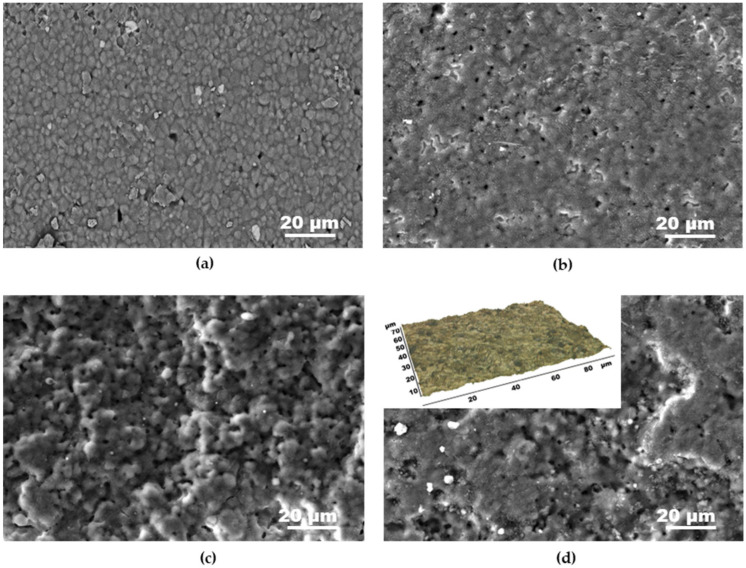
SEM images of the surface of AlN at different processing conditions: (**a**) the morphology of the native ceramic; (**b**) the laser fluence is 10.2 J/cm^2^ and the pulse overlapping is 6; (**c**) the laser fluence is 5.7 J/cm^2^ and the pulse overlapping is 6; and (**d**) the laser fluence is 10.2 J/cm^2^ and the pulse overlapping is 67. A 3D image from optical profilometer is also shown in (**d**).

The morphology characterization of the laser-processed areas reveals modifications of the surface compared to the native ceramic, as the relief characteristics depend on the experimental conditions. Figure 5 represents SEM images of the surface of AlN ceramic after treatment at different laser fluences and pulse overlapping. The native ceramic surface (Figure 5a) expresses a morphology defined by the AlN crystallites that compose the material. After the laser processing at the presented conditions, this morphology is not present and the ceramic surface seems covered by a thin melt layer. According to the performed analyses, it can be concluded that it is the conductive aluminum film formed after the laser-induced ceramic decomposition. Under the experimental conditions where the minimal resistance is obtained in this work (laser fluence of 10.2 J/cm^2^ and pulse overlapping of 6), the ceramic surface seems the smoothest one (Figure 5b). With the decrease in laser fluence to 5.7 Jcm^2^ and at the same overlapping, the surface morphology is characterized by the presence of a higher density of irregularities (Figure 5c).

Under these conditions, the resistance of the track is about 3 times higher compared to the minimal one, reaching a value of 52 Ohms. Figure 5d presents the morphology of the surface fabricated at a laser fluence of 10.2 J/cm^2^ and pulse overlapping of 67. The image reveals the formation of well-expressed micrometer irregularities with abrupt walls. The structure with this morphology is not conductive. These results indicate that the increase in the pulse overlapping may lead to ablation of big portions of material, which induces an interruption of the surface film discontinuity and leads to the formation of electrically nonconductive material. For a better representation of the surface morphology, 3D analysis of the surfaces was performed using an optical profilometer. An example of the obtained images in shown in Figure 5d. The analysis also gives the quantitative characterization of the surface roughness, as the obtained values for the average roughness (Ra) for the cases presented in Figure 5 are 0.5, 0.56, 2.56, and 3.8 µm for Figure 5a–d, respectively. It is found that the observed increase in the material resistance corresponds to the increase in the surface roughness expressed by Ra values.

**Figure 6:**
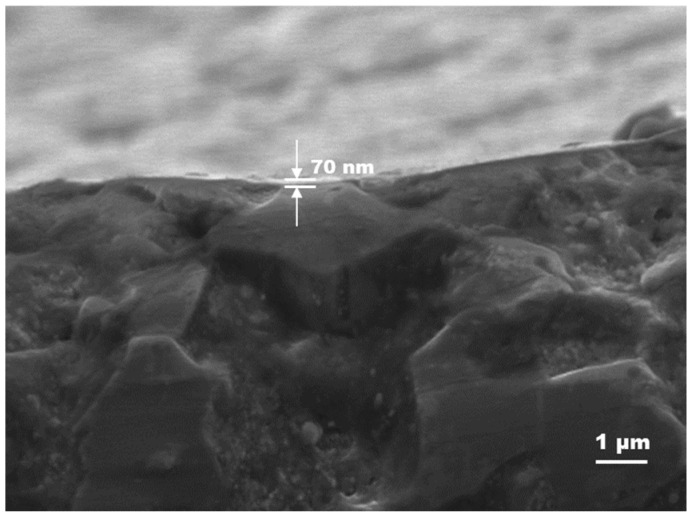
Cross-section SEM image of AlN ceramic processed at a laser fluence of 10.2 J/cm^2^ and pulse overlapping of 6. The analyzed sample was obtained by the breakage of the ceramic across the formed conductive track.

Using cross-sectional SEM imaging, the approximate thickness of the formed film on the ceramic surface could be estimated. Figure 6 shows an image for the material fabricated at the laser fluence of 10.2 J/cm^2^ and pulse overlapping of 6, where the track resistance is minimal. The sample for measurement was obtained by the breakage of the ceramic. Such an analysis indicates a thickness of the film of about 70 nm.

**Figure 7:**
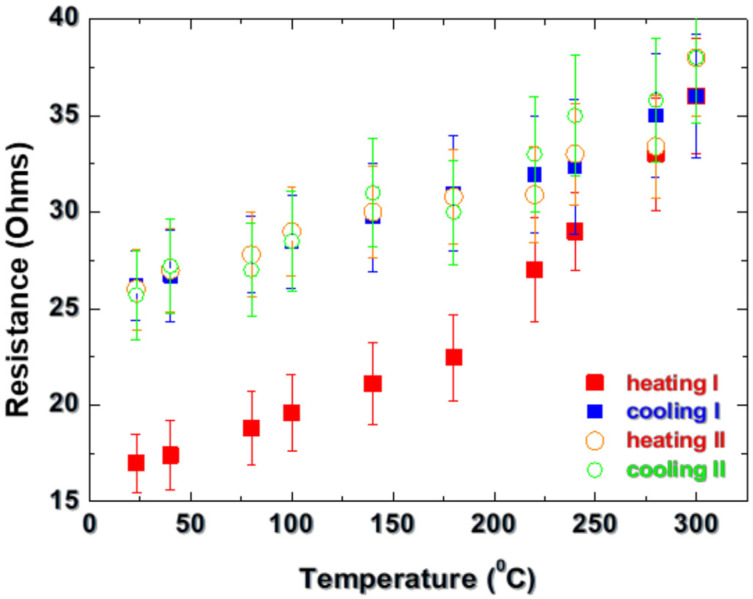
Dependence of the resistance of a track fabricated on AlN ceramic by laser processing at a fluence of 10.2 J/cm^2^ and pulse overlapping of 6 on the temperature for two cycles of heating and cooling. The statistics is based on measurement of three samples.

The further characterization of the fabricated conductive tracks includes an analysis of their thermal stability. Considering a potential use as heating element or operation at a higher temperature, this characteristic is crucial, since it will show the range of structure applicability. The first test is a “passive” one, where a conductive structure is heated by a heater and the resistance value is measured. The resistance measurement is also conducted in the cooling stage. Figure 7 represents the dependences for the structure obtained at a fluence of 10.2 J/cm^2^ and pulse overlapping of 6. Two consecutive tests were conducted, namely heating to a temperature of 300 °C (heating I), then cooling to room temperature (cooling I), and then performing the procedure again (heating II and cooling II). The maximal heating temperature is considered based on the fact that the oxidation of aluminum at temperatures lower than approximately 270 °C leads to the formation of an oxide layer with a thickness of about 2 nm, which is stable and prevents the metal from undergoing deeper oxidation [25]. Thus, in this temperature range the oxidation is expected to make a weak contribution to the track performance. The experiments indicate that the formed structure remains conductive for the used range. In the first heating experiment, the resistance increases with the temperature, as is expected for a metal. At temperatures of about 180 °C, a steeper dependence is observed (red squares). At a temperature of 300 °C, the resistance value reaches 36 Ohms. In the cooling stage, the resistance decreases more homogeneously as the steeper dependence is not observed (blue squares). The resistance value in all of the range remains higher than that for the heating stage, as at room temperature its value is 26 Ohms. The second run of heating (red circles) starts at this resistance value. In this case, a steeper increase in the resistance is not observed in the investigated temperature range. The resistance increases following a dependence that can be described well by a linear fit. In the cooling stage (blue circles), the resistance values are close to those in heating stage II for the corresponding temperature, with a maximal variation of about 10%.

**Figure 8:**
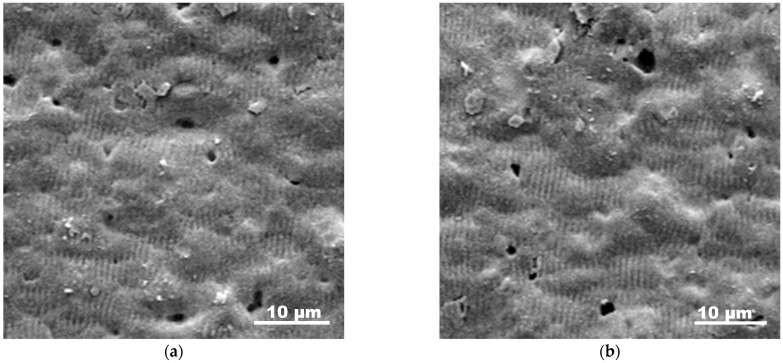
SEM images of the surface of a track fabricated in AlN at a fluence of 10.2 J/cm^2^ and pulse overlapping of 6: (**a**) before and (**b**) after annealing at 300 °C.

The presented behavior is an indication that in the first heating run a permanent change in the conductive material takes place. This could be related to aluminum layer morphology changes at micro- and nanoscales and/or an increase in the amount of the oxide phase. These changes, however, are permanent and lead to a stabilization of the structure, which results in a reversible behavior of the resistance with the temperature when repeating the experiment. Figure 8 represents SEM images of areas in a track fabricated at a fluence of 10.2 J/cm^2^ and pulse overlapping of 6, before (Figure 8a) and after (Figure 8b) the first run of heating and cooling. The structures express a characteristic ripple structure, which has been already reported for laser processing of AlN [26].

The presence of such a structure benefits analysis of the realization of phase changes as melting of the surface when such ripples could not be preserved. As seen in Figure 8, the surface morphology is preserved after heating, which is an indication that melting of the surface layer is not realized and the surface characteristics are stable after heating to 300 °C. Furthermore, application of macroscopic methods such as XPS and EDX does not show a clear change in the oxide phase. However, it should be considered that since the ceramic surface is rough, as can be seen in the SEM images, the conductive aluminum layer would have variation in its thickness. Since the melting or surface modification temperature of a thin film may depend on the thickness [27,28], it can be considered that some micro- or nanosized local changes could be realized, influencing the resistance. Since the steep increase in the resistance is not observed in the second heating experiment, it can be considered that these changes stabilize the morphology in terms of preserving its conductivity. The effect related to oxidation also could be taken into consideration, since even a small absolute increase in the oxide layer thickness could be significant as a relative value. In this case, however, one could consider a passivation effect.

It should be noted that for annealing at temperatures below approximately 150 °C, a steep increase in the structure resistance is not observed, and in the cooling stage the resistance values correspond to these in the heating stage at the given temperature, i.e., such a temperature does not cause a permanent modification of the material. Using the dependence as shown in Figure 7, one can estimate the temperature coefficient of resistance α from the relation of the change in the resistance, R, with the temperature change ΔT, R = R_0_ (1 + αΔT) [29]. An estimation using the dependence presented in Figure 7 for heating II gives a value of 1.4 × 10^−3^ °C^−1^, which is about 3 times lower than that for aluminum. This could be explained by the more complex composition of the formed conductive layer (the presence of AlN, Al_5_Y_3_O_12_) and the observed decrease in α with the film thickness [30].

**Figure 9:**
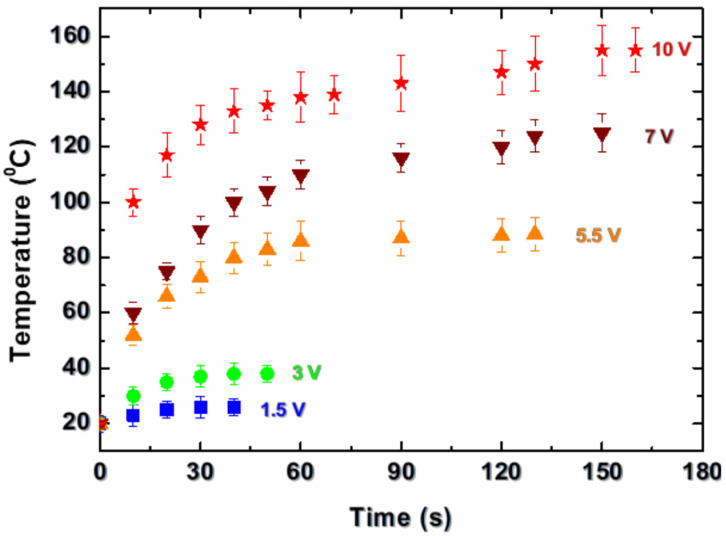
Dependences of the temperature as a function of time for different DC voltages applied to track formed in AlN ceramic at a laser fluence of 10.2 J/cm^2^ and pulse overlapping of 6.

In the second type of experiments, the laser-fabricated structures were used as a heating element. For this purpose, DC voltage was applied between the two electrodes located at the two ends of the track (see Figure 1). Figure 9 shows the dependence of the temperature on the time when a different voltage was applied to an element obtained with a laser fluence of 10.2 J/cm^2^ and pulse overlapping of 6. The sample was annealed to 300 °C under the conditions presented in Figure 7 (first cycle). The dependencies are traced until the change in the temperature is about 1 °C per 10 s. The resistance value for the temperature range achieved at 10 V changes from 26 to about 28 Ohms, which defines an average current of about 370 mA through the track. The stability of the heater element is traced for a period of 2 h for the cases of application of 5.5 V and 10 V. In the first case, the element supports the temperature for the measuring time. This experiment was repeated three times, and the results for the achieved maximal temperature differ by less than 10%. In the case of operation at 10 V, the temperature is stable for a period of about 30 min then the resistance increases rapidly, with a jump of about an order of magnitude (230 Ohms), which results in a current drop. As a result, the maximal temperature decreases to about 50 °C and stays stable for the rest of the measuring time. Although the resistance value decreases in the cooling stage, it remains about an order of magnitude higher after the cooling of the sample to the room temperature. When the sample is cooled, it is found that a slight movement in the electrodes results in a return of the resistance value to the initial value of about 26 Ohms. This is an indication that a change in the material in the contact area between the electrodes and the structure could be responsible for the observed behavior while the rest of the material in the track preserves its properties. In the contact zone, the contact resistance could be higher due to the small contact area between the track having a high roughness, as seen in the SEM images, and the electrodes.

**Figure 10:**
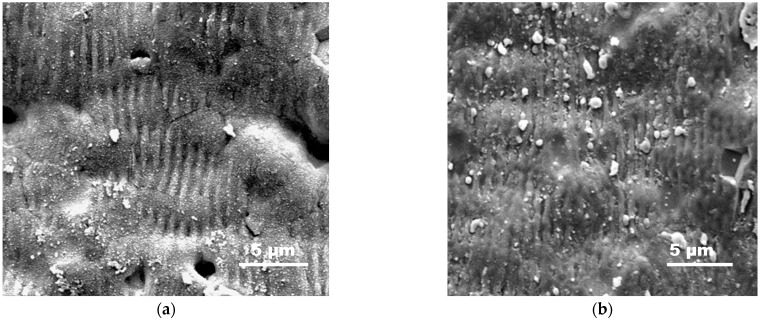
SEM images of areas from a conductive track located approximately in the middle of track (**a**) and in the zone of contact between one of the electrodes and track (**b**). The sample is prepared by laser processing at a laser fluence of 10.2 J/cm^2^ and pulse overlapping of 6; then it is annealed to 300 °C following the procedure described in Figure 7, and used as a heater applying 10 V for 2 h.

The increased contact resistance could induce local heating of the material, leading to enhanced oxidation and material modification, which may result in the conductivity being cut off, as the effect is more pronounced with the increase in the current. In order to clarify a change in the material, the morphology of the area of contact was examined. Figure 10 shows SEM images of an area approximately in the middle of the processed area and in the zone of the contact between one of the electrodes and the track formed. The fabrication conditions are the same as in Figure 9 and 10 V is applied on the sample. The areas of contact can be easily defined since they express a light brown color visible with the naked eye. A comparison of the images shows that in the contact area (Figure 10b), the characteristic ripples are observed in fewer areas on the surface and they express smoother edges. The effect can be attributed to locally enhanced heating that induced melting or softening of the material, which blurs the crests’ borders and can be induced by a high contact resistance. Furthermore, EDX analysis indicates an increase in the presence of oxygen of about 12% in the contact zone, compared to the middle of the track. Thus, an increase in the oxide layer in the zone is realized.

Based on the results obtained, it can be concluded that, under the presented conditions and experimental setup, the laser-induced tracks in AlN ceramic can be used as an integrated heating element that can be used reproducibly to a maximal temperature of about 90 °C (current of about 200 mA). The heater performance could be improved by engineering solutions to achieve better connection of the electrodes, for example, by soldering or other bonding technologies. Furthermore, encapsulation of the structure to prevent oxidation could enable a use for higher temperatures. The main benefits of the demonstrated structures are easy fabrication of the conductive tracks; excellent integration into the ceramic, which is a material with high thermal conductivity, allowing fast spreading of heat in a large area; and the ability to fabricate small heating elements and those with a more complex shape defined by laser scanning on the desired geometry. Furthermore, the proposed method of laser-induced conductive track formation could offer some advantages compared to commercial technologies such as printing. In laser technology, no additional material such as ink is needed and the adhesion of the formed material is very good. In fact, there is no clear border between the formed aluminum layer and the ceramic, but the transition is smooth. This makes the conductive layer very stable, even for mechanical interaction. The laser method offers a change in the track resistance in a broad range by changing the processing parameters. This could be useful for the design of heaters with a small size or an electric circuit that includes AlN ceramic. In addition, the fabrication method does not require initial preparation of the ceramics surface. The conductive structures on ceramic can be formed on the ceramic as it is delivered by the producer. The reason for this is that the laser processing removes the surface layer. Since the laser radiation can be focused at a spot with a size less than the used wavelength, even sub-micrometer tracks could be fabricated. This increases the spatial resolution of the method. Furthermore, if for any reason the conductive track is damaged, it can be easily re-fabricated by the same process on the same place.

## 4. Conclusions

In this work, a novel application of laser-induced conductive tracks in AlN is presented. It considers the development of an integrated heating element. The conductive material is formed due to thermal decomposition of the ceramic, which leads to the formation of an aluminum layer. The influence of the temperature on the electrical properties of the formed structures is revealed and discussed. It is found that the conductive tracks can be stabilized by temperature annealing, which results in an increase in the initial resistance, but ensures predictable behavior of this parameter in a subsequent heating. Such behavior is confirmed under temperatures of up to 300 °C. For this temperature interval, the temperature coefficient of resistance is about 3 times lower compared to that of aluminum. The conductive tracks can be used as a resistive heater because, without considering measures to prevent oxidation and using a simple introduction of electrodes, it can support reliable heating of a 1 cm^2^ AlN ceramic slab to about 90 °C at an operation voltage of 5.5 V. Operation at higher temperatures is also demonstrated, as the maximal temperature achieved is about 150 °C at 10 V. Applying higher voltages results in a modification of the material at the contact between the electrodes and the formed track, which leads to an increase in the resistance and drop in the temperature. Since this change is related only to the contact area, it can be reduced by a different type of electrode attachment. The main benefits of this heater, in terms of easy fabrication, direct integration of the heating element (conductive line) into ceramic, and the ability to easily fabricate elements with different sizes and shapes that offer laser processing, can be a basis for the design and efficient development of an integrated heating element with applications in different areas.

## Data Availability

More data supporting the reported results can be given upon request.
